# The Pheromone-Regulated Membrane Protein CsPRM10 Plays an Essential Role in the Asexual Reproduction of the Pepper Anthracnose Fungus *Colletotrichum scovillei*

**DOI:** 10.3390/jof12020086

**Published:** 2026-01-27

**Authors:** Haowei Shen, Jiaping Li, Wenjie Xu, Guoyang Gao, Kyoung Su Kim, Jian-Xin Deng, Teng Fu

**Affiliations:** 1 Department of Plant Protection, Engineering Research Center of Ecology and Agricultural Use of Wetland, and MARA Key Laboratory of Sustainable Crop Production in the Middle Reaches of the Yangtze River, Yangtze University, Jingzhou 434025, China; blank749123@outlook.com (H.S.); ljpchn13471076758@163.com (J.L.); 201504436@yangtzeu.edu.cn (W.X.); djxin555@yangtzeu.edu.cn (J.-X.D.); 2 Division of Bio-Resource Sciences, Interdisciplinary Program in Smart Agriculture, and Bioherb Research Institute, Kangwon National University, Chuncheon 24341, Republic of Korea; ggy1@kangwon.ac.kr (G.G.); kims@kangwon.ac.kr (K.S.K.)

**Keywords:** *Colletotrichum scovillei*, pheromone-regulated membrane protein, conidiation, surface hydrophobicity

## Abstract

The phytopathogenic fungus *Colletotrichum scovillei* causes a destructive anthracnose on pepper fruit worldwide. Conidiation plays an essential role in the dissemination of pathogenic fungi, yet the regulatory mechanisms underlying this process remain largely unknown. In this study, a pheromone-regulated membrane protein 10 (PRM10) was identified in *C. scovillei*, whose function has not been characterized in fungal plant pathogens previously. The targeted gene deletion mutant (*ΔCsprm10*) was normal in plant infection but showed a decrease in surface hydrophobicity compared to the wild-type strain. Notably, *ΔCsprm10* was completely defective in conidiation. A microscopic observation further confirmed that *ΔCsprm10* failed to form conidiophores, suggesting that *CsPRM10* plays an essential role in the conidiation of *C. scovillei* by regulating conidiophore development. The transcriptomic analysis indicated that the loss of *CsPRM10* caused differential expressions of genes related to membrane-associated processes and nuclear functions. Taken together, these findings suggest that *CsPRM10* acts as a novel regulator of conidiation in *C. scovillei* and provide new insights into the molecular basis of fungal asexual development.

## 1. Introduction

Anthracnose caused by *Colletotrichum* species is a destructive disease occurring on many agriculturally important crops, vegetables, and fruits, leading to a considerable economic loss around the world annually [1,2,3]. *Colletotrichum scovillei* in the *Colletotrichum acutatum* species complex causes anthracnose on pepper fruits during preharvest and postharvest storage in many countries, especially those in East and Southeast Asia [3,4]. The infection of *C. scovillei* starts with conidia landing on and adhering to the surface of pepper fruits [5]. With the perception of host physical and chemical signals, conidia germinate and differentiate appressoria at the tips of germ tubes. Subsequently, the appressoria develop tiny pegs that penetrate and form highly branched hyphae visualized as dendroid structures inside the cuticle layer of pepper fruits [6]. Following the successful infection of fruit tissue, typical anthracnose lesions are formed, and abundant pinkish conidia are produced, which serve as the main inoculum for disease epidemics [5,7].

Conidiation is a critical process for the dissemination of plant pathogenic fungi [8,9]. Inhibiting conidiation has been considered a promising strategy to control fungal disease epidemics [10,11]. Although a number of commercial fungicides are effective in suppressing fungal growth and host infection, those specifically targeting conidiation remain undeveloped [12,13,14]. Conidiation is a highly regulated developmental process involving a series of complex cellular and morphological events. It is initiated by the formation of aerial hyphae, which subsequently differentiate into conidiophores [6,9,15]. The apical cells of conidiophores further develop into phialides, which ultimately produce conidia [16,17]. Unlike the saprotrophic filamentous fungus *Aspergillus nidulans*, the molecular mechanisms underlying the conidiation of plant pathogenic ascomycete fungi remain largely unexplored, and only a few genes have been identified as being associated with this process. For example, a T-DNA insertion mutant of *COS1* was completely defective in conidiophore development in *Magnaporthe oryzae* [18]. The *MoHOX2* deletion mutant could develop conidiophores but failed to produce conidia [15]. *MoMsn2* promotes the differentiation of aerial hyphae into conidiophores of *M. oryzae*, and its deletion mutant showed a severe reduction in conidiation [19]. In *Fusarium graminearum*, the deletion of *StuA*, encoding an APSES transcription factor, resulted in severe defects in conidiophore development and conidiation [20]. The deletion of *FgYCK1* blocked phialide development, thereby abolishing conidiation [21]. In the pepper anthracnose fungus *C. scovillei*, the transcription factors CsHOX2 and CsCZF9 were found to regulate conidia production but not conidiophore development [5,6].

The pheromone-regulated membrane protein 10 (PRM10) was first described as a stress response protein in *Saccharomyces cerevisiae* [22]. Subsequent studies have mainly focused on its transcriptional responses to stress conditions. For example, the *PRM10* of *S. cerevisiae* was upregulated by exogenous treatments of mating pheromones (α-factors), the β-1,3-glucan synthase inhibitor (caspofungin), or sugar-induced osmotic stress and the overexpression of protein kinase C, small GTPase Rho2, or transcription factor Ste12 [23,24]. Furthermore, a point mutation in PRM10 enhanced the uptake of mevalonate in *S. cerevisiae* [25]. Recently, the *PRM10* ortholog was found to be highly expressed in the basidiospores of *Puccinia striiformis* f. sp. *tritici*, the causal agent of wheat stripe rust [26]. However, the functional roles of *PRM10* remain uncharacterized in plant pathogenic fungi.

In this study, we aimed at investigating the functions of *CsPRM10*, orthologous to *S. cerevisiae PRM10*, in the fungal differentiation and pathogenicity of *C. scovillei* by using the deletion mutant (*ΔCsprm10*). *ΔCsprm10* was found to be completely defective in conidiation on both artificial medium and the host plant. The defect found in *ΔCsprm10* was rescued in a complemented strain (*Csprm10c*). Pathogenicity assay showed that *ΔCsprm10* was normal in plant infection, compared to the wild-type and *Csprm10c* strains. Collectively, our results suggest that *CsPRM10* plays a fundamental role in the conidiation of *C. scovillei*.

## 2. Materials and Methods

### 2.1. Fungal Strains and Culture Conditions

The *C. scovillei* strain KC05 was used as the wild-type strain in this study [27]. All strains were cultured on V8 agar (V8A), minimal medium agar (MMA), potato dextrose agar (PDA), complete medium agar (CMA), and oatmeal medium agar (OMA) in a growth chamber, as previously described [28].

### 2.2. Bioinformatic Analysis

The sequences of CsPRM10 homologs were retrieved from the National Center for Biotechnology Information (NCBI, https://www.ncbi.nlm.nih.gov/, accessed on 10 April 2025). Phylogenetic analysis was conducted using MEGA12 (version 12.0.11). The domains of CsPRM10 homologs were predicted using InterProScan.

### 2.3. Generation of Knock-Out and Complemented Strains

Genomic DNA was extracted following previously reported methods [27,29]. The *CsPRM10* deletion construct was generated using the double-joint PCR [30]. The upstream and downstream regions of *CsPRM10* were amplified using paired primers PRM10_5F/R and PRM10_3F/R, respectively, while the hygromycin resistance cassette (*HPH*) was amplified using paired primers HPH_F/R (Table S1). These three amplified fragments were fused by PCR with primers PRM10_5F/3R, and the fused fragments were amplified with primers PRM10_NF/NR (Table S1). The resulting constructs were transformed into protoplasts of the wild-type strain following a previous method [28]. The knock-out mutants growing on TB3 agar were first selected by a screening PCR using paired primers PRM10_SF/SR (Table S1) and then confirmed through Southern blotting. To construct the complemented strain, protoplasts of *ΔCsPRM10* were transformed with the full length of *CsPRM10* together with the geneticin resistance cassette. Complemented strains were verified through a reverse transcription (RT) PCR.

### 2.4. Analyses of Fungal Growth and Developments

To evaluate mycelial growth, mycelial agar plugs obtained from three-day-old MMA were inoculated to V8A, MMA, PDA, and CMA and cultured in a growth chamber at 25 °C and in darkness [31]. After six days, photographs of cultured mycelia were taken, and colony diameters were measured. To evaluate fungal stress adaptation, strains were grown on CMA containing 300 ppm Congo red (CR) and 0.005% sodium dodecyl sulfate (SDS) at 25 °C and in darkness, and the colony diameters were measured, and photographs were taken after five days. The surface hydrophobicity of mycelia was assessed by dropping sterile distilled water (SDW) and 0.1% SDS solution onto mycelia grown on PDA, CMA, and MMA. After 1 min, photographs were taken. To evaluate conidiation, conidia were harvested with 5 mL SDW from V8A and PDA and 3 mL SDW from seven-day-old lesions of pepper fruit and counted using a hemocytometer (Marienfeld-Superior, Berlin, Germany). To visualize the conidiophores, mycelial agar plugs obtained from three-day-old MMA were inoculated to OMA and cultured at 25 °C and in darkness for four days. Then, the obtained mycelial agar plugs from OMA were transferred to a humid box and cultured at 25 °C and in lightness for five hours. Aerial hyphae and conidiophores were stained with a modified lactophenol–aniline blue solution, washed with SDW, and observed under a light microscope. To visualize appressorium-like structures (ALSs), mycelial agar plugs from four-day-old OMA were inoculated onto the hydrophobic surface of coverslips placed inside a humid box and cultured at 25 °C and in darkness for five days. All experiments were independently conducted three times with at least three replicates each time.

### 2.5. Pathogenicity Assays

Mycelial agar plugs (5 mm in diameter), which did not contain any conidium, were obtained from four-day-old OMA. Artificial wounds were created on intact pepper fruits by piercing the pearl of intact fruits with a syringe needle (0.6 mm in diameter). Both artificially wounded and intact pepper fruits were inoculated with the mycelial agar plugs described above and incubated in a humid chamber at 25 °C. Photographs of lesions on wounded and intact pepper fruits were taken after six and ten days, respectively. The relative lesion size was estimated using ImageJ (version 1.53t). The pathogenicity assay was performed in three independent experiments. In each experiment, three pepper fruits were used for the treatment of the wild-type, *ΔCsprm10*, and *Csprm10c* strains, and each fruit was inoculated with two mycelial agar plugs.

### 2.6. RNA Sequencing

Fresh fungal mycelia grown in CM broth were transferred to V8 broth to induce conidiophore differentiation. Fungal tissues were separated using 3 layers of Miracloth and immediately frozen in liquid nitrogen for RNA isolation. Three biological replicates of extracted RNA were sequenced on an Illumina platform. A total of 45.8 Gb of clean data was obtained and mapped to the published genome (https://doi.org/10.6084/m9.figshare.26112565) using HISAT2 (version 2.2.1) [5]. Differentially expressed genes (DEGs) between the *ΔCsprm10* and wild-type strains were analyzed using DESeq2 (version 1.36.0) [32]. Genes with an adjusted *p* value (false discovery rate, FDR) < 0.05 and |log_2_ fold change| > 1 were considered differentially expressed.

## 3. Results

### 3.1. Phylogenetic Analysis of CsPRM10

**Figure 1:**
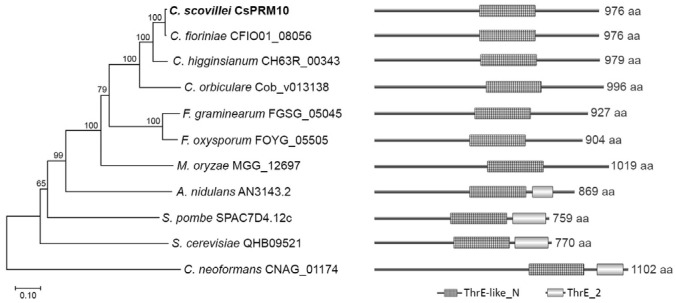
Phylogenetic analysis and domain prediction. The phylogenetic tree (left panel) was constructed by using the neighbor-joining method with 1000 bootstraps. Domains, including ThrE-like_N (IPR010619) and ThrE_2 (IPR024528), were predicted using InterPro. The full-length amino acid sequences of CsPRM10 orthologs were used to conduct the phylogenetic analysis and domain prediction.

*CsPRM10* is predicted to encode a protein containing 976 amino acids. To determine the relationship between CsPRM10 and its homologs, we performed a phylogenetic analysis and domain prediction. The phylogenetic analysis showed that CsPRM10 is closely related to its orthologs from *Colletotrichum* spp. and distantly related to its ortholog in a human pathogen *Cryptococcus neoformans* (Figure 1). Domain prediction showed that all CsPRM10 orthologs contain a ThrE-like_N (threonine/serine exporter-like, N-terminal domain) domain, while only the CsPRM10 orthologs from *A. nidulans*, *Schizosaccharomyces pombe*, *Saccharomyces cerevisiae*, and *C. neoformans* have an additional domain, threonine/serine exporter, ThrE (threonine/serine exporter, ThrE) (Figure 1). These results suggest that CsPRM10 orthologs are conserved in plant pathogenic fungi.

### 3.2. Targeted Deletion of CsPRM10

**Figure 2:**
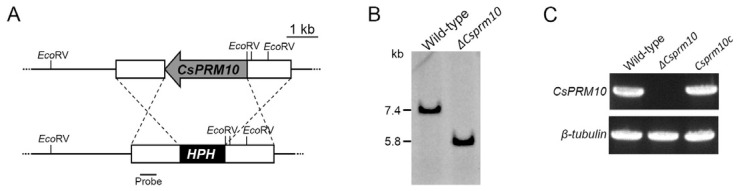
Generation and verification of deletion mutant. (**A**) Targeted deletion of *CsPRM10*. Homologous recombination was used to replace *CsPRM10* with HPH cassette. (**B**) Confirmation of *CsPRM10* deletion by Southern blotting. Genomic DNA was digested with EcoRI and hybridized to biotin-labeled probe. (**C**) Expression of *CsPRM10*. RT-PCR was used to detect expression of *CsPRM10* in indicated strains, and β-tubulin was used as endogenous control.

To investigate the functions of *CsPRM10*, we generated a deletion mutant by using homologous recombination (Figure 2A). The resulting deletion mutant was verified through Southern blotting (Figure 2B). Genomic copies of *CsPRM10* were re-transformed into *ΔCsprm10* to generate a complemented strain, *Csprm10c*. As expected, the sequence within *CsPRM10* was detected in the wild-type strain and *Csprm10c* but not in *ΔCsprm10* by RT-PCR (Figure 2C).

### 3.3. CsPRM10 Is Involved in Surface Hydrophobicity

**Figure 3:**
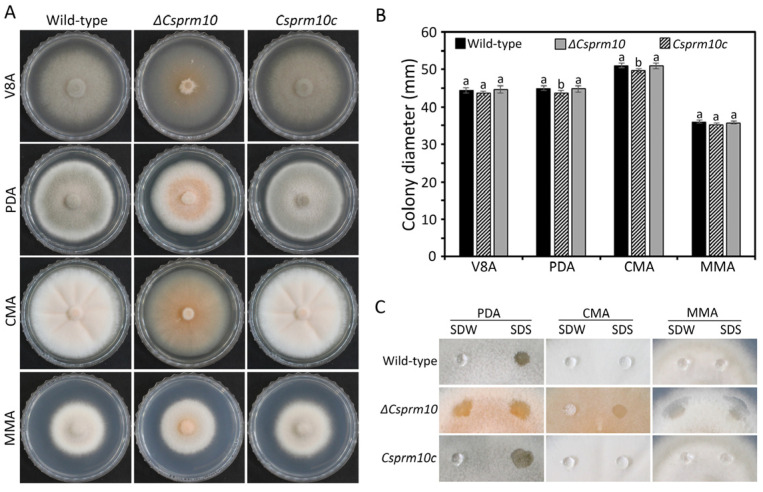
Mycelial growth and surface hydrophobicity. (**A**,**B**) Mycelial growth. Mycelial agar plugs from three-day-old MMA were inoculated to V8A, PDA, CMA, and MMA and cultured at 25 °C and in darkness. (**A**) Photographs of mycelial growth. (**B**) Measurement of colony diameters. (**C**) Surface hydrophobicity. Drops of SDW and 0.1% SDS were placed on mycelia and incubated for 30 s. The different lowercase letters in a group indicate significant difference estimated using Duncan's test (*p *< 0.05).

Mycelial growth was evaluated by measuring the colony diameters. The colony diameters of *ΔCsprm10* were similar on V8A and MMA and slightly reduced on PDA and CMA, compared to those of the wild-type and *Csprm10c* strains (Figure 3A,B). Interestingly, the mycelia of *ΔCsprm10* were found to be more wettable than those of the wild-type and *Csprm10c* strains on V8A and CMA. To test whether *CsPRM10* is involved in the surface hydrophobicity of *C. scovillei*, drops of SDW and SDS were placed on mycelia. The result showed that SDW or SDS remained beaded on the mycelia of the wild-type and *Csprm10c* strains. However, the mycelia of *ΔCsprm10* were easily soaked by SDW or SDS. This result suggests that *CsPRM10* is involved in the surface hydrophobicity of *C. scovillei*.

### 3.4. CsPRM10 Is Essential for Conidiation

**Figure 4:**
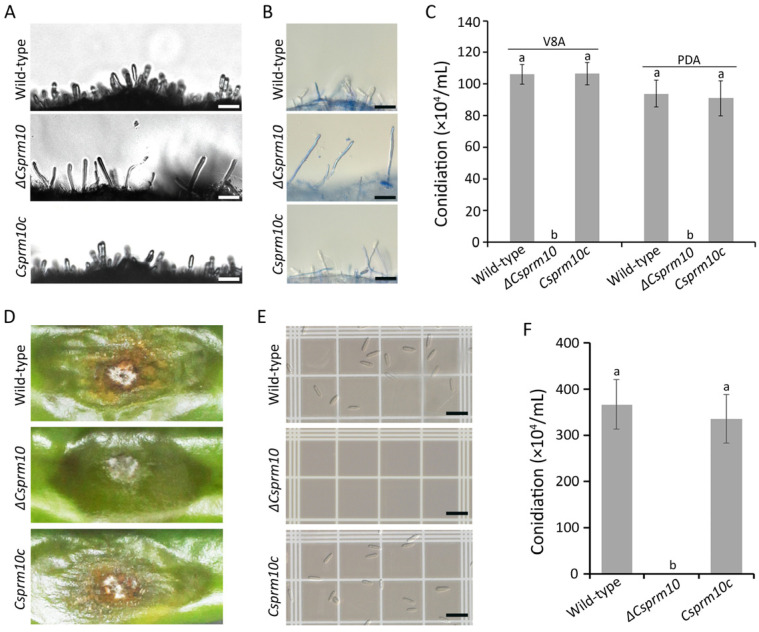
Conidiation. (**A**–**C**) Evaluation of conidiation on artificial medium. (**A**) Microscopic observation of conidiation on OMA. (**B**) Mycelia were stained with blue color using lactophenol–aniline blue staining. (**C**) Quantitative measurement of conidiation on V8A and PDA. (**D**–**F**) Evaluation of conidiation on lesion of host fruit. (**D**) Photographs of conidia masses on anthracnose lesions. (**E**) Conidia formed on lesions were washed with 3 mL SDW and observed using light microscope. (**F**) Quantitative measurement of conidiation on anthracnose lesions. Scale = 15 µm. The different lowercase letters in a group indicate significant difference estimated using Duncan's test (*p *< 0.05).

Microscopic observation showed that the wild-type and *Csprm10c* strains formed many conidia, whereas *ΔCsprm10* failed to produce any conidia (Figure 4A). To determine whether *ΔCsprm10* could form conidiophores, lactophenol–aniline blue was used to identify conidiophores by staining aerial hyphae. The result showed that *ΔCsprm10* was unable to form conidiophores, compared to many conidiophores observed in the wild-type and *Csprm10c* strains (Figure 4B). We next performed a quantitative analysis of conidiation on V8A and PDA. Expectedly, *ΔCsprm10* was completely defective in conidiation, whereas the wild-type and *Csprm10c* strains produced many conidia (Figure 4C). To further investigate whether *CsPRM10* is related to conidiation on the host plant, we counted conidia on anthracnose lesions of pepper fruit. The result showed that anthracnose lesions caused by the wild-type and *Csprm10c* strains formed orange conidium masses, whereas only white mycelia were observed on anthracnose lesions caused by *ΔCsprm10* (Figure 4D,E). Quantitative analysis showed that *ΔCsprm10* did not produce any conidia, whereas the wild-type and *Csprm10c* strains formed (367.2 ± 53.4) × 10^4^ conidia/mL and (336.1 ± 52.1) × 10^4^ conidia/mL, respectively (Figure 4F). These results suggest that *CsPRM10* is essential for the conidiation and conidiophore development of *C. scovillei*.

### 3.5. CsPRM10 Is Associated with Stress Adaptation

**Figure 5:**
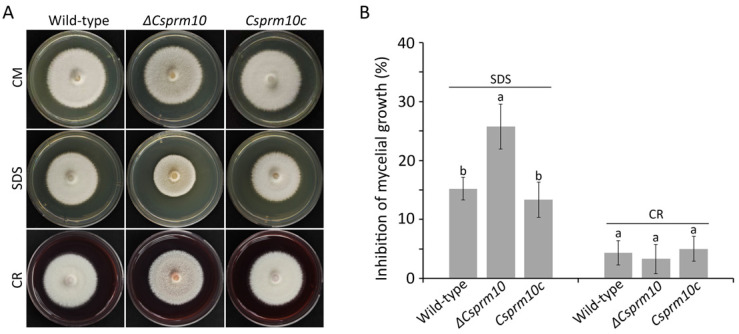
Stress adaptations. Mycelial agar plugs from 3-day-old MMA were inoculated to CMA with or without sodium dodecyl sulfate (SDS) and Congo red (CR) and cultured for 5 days. (**A**) Photographs of mycelial growth. (**B**) Evaluation of inhibition of colony growth. The different lowercase letters in a group indicate significant difference estimated using Duncan's test (*p *< 0.05).

To test whether *CsPRM10* is involved in adaptation to cell wall and membrane stresses, we evaluated the colony growth of *ΔCsprm10* on CMA amended with CR and SDS. The result showed that the mycelial growth of *ΔCsprm10* was significantly inhibited by SDS but not CR compared to the wild-type (Figure 5A,B). The defect of *ΔCsprm10* in response to SDS was restored in *Csprm10c* (Figure 5A,B). This result suggests that *CsPRM10* is involved in tolerance to cell membrane stress.

### 3.6. CsPRM10 Is Dispensable for Disease Development

**Figure 6:**
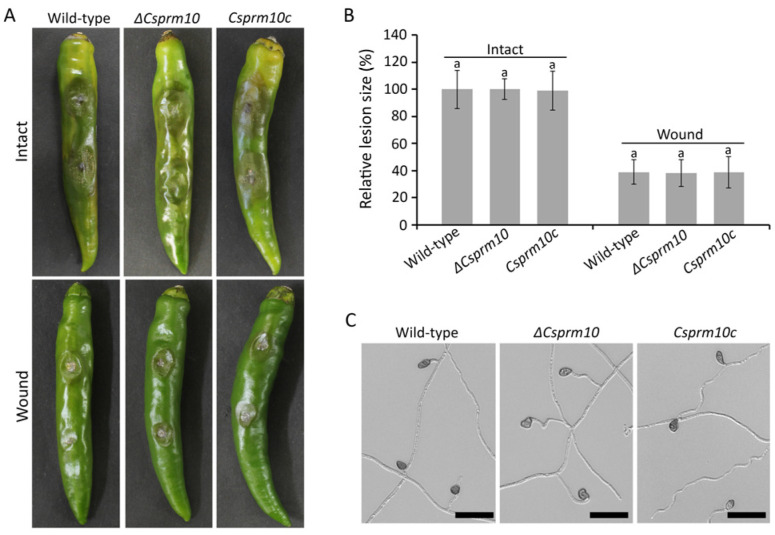
Pathogenicity assays. (**A**,**B**) Evaluation of disease severity. (**A**) Mycelial agar plugs from 4-day-old OMA were inoculated to intact and wounded pepper fruits and incubated for 10 and 6 days, respectively. (**B**) Size of anthracnose lesion was quantitatively measured using Image J. Anthracnose lesion caused by wild-type strain on intact fruit was calculated as a relative of 1. (**C**) Appressorium-like structure (ALS) formation. Mycelial agar plugs from 4-day-old OMA were inoculated onto hydrophobic surface of coverslips and incubated in humid box for 5 days. Scale = 20 µm. The same lowercase letters in a group indicate no significant difference estimated using Duncan's test (*p *< 0.05).

As *ΔCsprm10* is completely defective in conidiation, we performed pathogenicity assay by inoculating mycelial agar plugs onto healthy pepper fruits. The result showed that all intact and wounded pepper fruits inoculated with *ΔCsprm10* developed typical anthracnose (Figure 6A), and the lesion size was comparable to those observed in fruits inoculated with the wild-type and *Csprm10c* strains (Figure 6B). We further evaluated ALS formation by inoculating mycelial agar plugs onto the hydrophobic surface of cover glass. *ΔCsprm10* was found to form ALSs similar to the wild-type and *Csprm10c* strains (Figure 6C). These results suggest that *CsPRM10* is dispensable for the pathogenicity of *C. scovillei*.

### 3.7. Transcriptomic Profiling of ΔCsprm10

**Figure 7:**
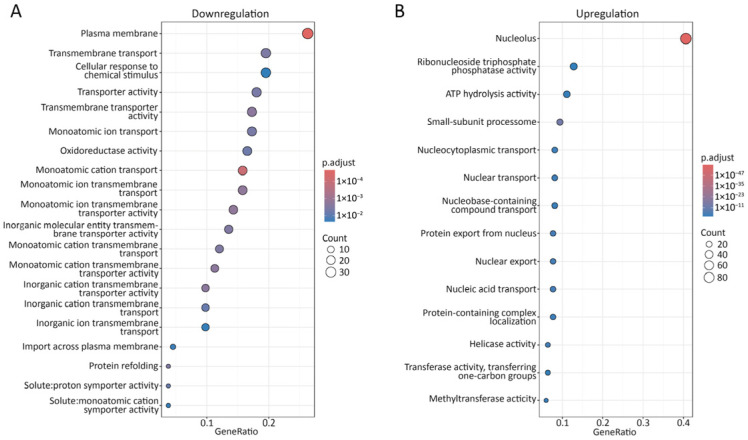
Gene ontology (GO) enrichment analysis. Differentially expressed genes (DEGs) were obtained from RNA-seq analysis. Downregulated (**A**) and upregulated (**B**) DEGs were classified based on GO terms.

To investigate the transcriptomic response following *CsPRM10* deletion, an RNA-seq analysis was conducted to profile the gene expressions of *ΔCsprm10*, compared to the wild-type strain. This analysis identified 1968 DEGs (*p* < 0.05), with 1199 downregulated DEGs (log2 fold change < −1) and 769 upregulated DEGs (log2 fold change > 1) (Figure S1, Table S2). Gene Ontology (GO) enrichment analysis showed that the downregulated DEGs were enriched in membrane-associated GO categories, including plasma membrane, transmembrane transport, and transmembrane transporter activity (Figure 7A). Moreover, multiple transport-related biological processes were overrepresented, such as monoatomic ion transport, cation transmembrane transport, and import across the plasma membrane (Figure 7A). In contrast, upregulated DEGs were predominantly enriched in GO terms related to nuclear and nucleocytoplasmic processes, including nucleolus, nuclear transport, nucleocytoplasmic transport, and protein export from nucleus (Figure 7B). Several molecular function categories linked to energy-dependent and nucleic acid-associated activities, such as ATP hydrolysis activity, helicase activity, and ribonucleoside triphosphate phosphatase activity, were also overrepresented (Figure 7B). Together, these results indicate distinct GO enrichments between downregulated and upregulated DEGs in *ΔCsprm10*, with membrane-related transport processes represented among downregulated DEGs and nuclear-associated functions enriched among upregulated DEGs.

## 4. Discussion

In this study, we functionally characterized *CsPRM10* in the pepper anthracnose fungus *C. scovillei*. Phylogenetic analysis indicated that CsPRM10 is closely related to its homologs from filamentous plant pathogenic fungi. Consistent with this evolutionary divergence, domain prediction showed that CsPRM10 homologs from plant pathogenic fungi harbor a ThrE-like_N domain but lack the ThrE domain (Figure 1). This domain architecture probably reflects an evolutionary adaptation of CsPRM10 to fungal differentiation and virulence in plant pathogenic fungi.

A major finding of this study is that the deletion of *CsPRM10* resulted in a complete loss of conidiation in *C. scovillei* (Figure 4C,F). Microscopic examination further confirmed that *ΔCsprm10* failed to form conidiophores, indicating that *CsPRM10* plays a critical role at an early stage of conidiation. These data clearly distinguish *CsPRM10* from previously characterized conidiation-related regulators in *C. scovillei*, such as *CsHOX2* and *CsCZF1*, that are essential for conidium production but dispensable for conidiophore formation [5,6]. Therefore, *CsPRM10* represents another class of developmental regulator required for the establishment of conidiophore architecture, rather than conidium generation. *CsPRM10* was also found to be involved in the regulation of surface hydrophobicity, as evidenced by the mycelia of *ΔCsprm10* displaying increased wettability compared to the wild-type strain (Figure 3C). Surface hydrophobicity has been widely recognized as a key determinant of fungal development and is required for the emergence of aerial hyphae, conidiophore formation, and conidium dispersal [33,34,35]. The decreased surface hydrophobicity of *ΔCsprm10* suggests that *CsPRM10* may be involved in membrane-associated functions that regulate cell wall assembly and hydrophobin deployment, thereby enabling the formation of aerial hyphae and conidiophores.

The RNA-seq analysis conducted at an early stage of conidiophore development revealed the altered expression of genes associated with membrane-related processes and nuclear functions, providing a mechanistic insight for the failure of conidiophore formation in the absence of *CsPRM10* (Figure 7). Genes downregulated in *ΔCsprm10* were predominantly enriched in membrane-associated transport processes, including transmembrane transport and ion transport. Proper membrane organization and solute transport are known to be involved in polarized growth and cellular differentiation during conidiophore development [36,37]. Notably, the ortholog of *flbC* was identified among the downregulated genes. *flbC* has been documented as an important regulator of aerial hyphal development and conidiation in *A. nidulans* and *M. oryzae* [38,39]. In addition, several genes encoding cell wall- and surface-associated proteins were downregulated, including those predicted to encode hydrophobins and chitin synthases. In filamentous fungi, hydrophobin and chitin synthase have been implicated in aerial hyphal formation and conidiation [35,40,41,42].

Despite its roles in regulating conidiation and surface hydrophobicity, the deletion of *CsPRM10* did not affect the pathogenicity of *C. scovillei* (Figure 6A,B). This observation contrasts with previous studies which have reported a close relationship between surface hydrophobicity and fungal pathogenicity. Defects in surface hydrophobicity often lead to impaired host recognition and compromised host infection [43,44]. Considering that *C. scovillei* forms dendroid structures within the cuticle layer of pepper fruit [6], *C. scovillei* may employ alternative mechanisms, such as chemical signaling and enzymatic degradation at an early stage of host plant infection. In addition, *ΔCsprm10* retained the ability to form ALSs at levels comparable to those of the wild-type strain (Figure 6C), suggesting that the differentiation of infection-related structures in *C. scovillei* is largely independent of surface hydrophobicity.

## Data Availability

The transcriptomic data presented in the study are openly available in CNCB at CRA036200.

## Supplementary Materials

The following supporting information can be downloaded at https://www.mdpi.com/article/10.3390/jof12020086/s1, Figure S1: Global analysis of RNA-seq data. (A) Density of fragments per kilobase of transcript per million mapped reads (FPKM). (B) Correlation analysis among six samples. (C) Volcano diagram of differentially expressed genes in *∆Csprm10* referenced to wild-type strain; Table S1: Primers used in this study; Table S2: List of differentially expressed genes.

## Abbreviations

The following abbreviations are used in this manuscript:

DEGs	Differentially expressed genes
GO	Gene Ontology
V8A	V8 agar
MMA	Minimal medium agar
CM	Complete medium agar
PDA	Potato dextrose agar
CR	Congo red
SDS	Sodium dodecyl sulfate
SDW	Sterile distilled water
ALS	Appressorium-like structure
